# Extraction and Characterization of Tannins from the Barks of Four Tropical Wood Species and Formulation of Bioresins for Potential Industrial Applications

**DOI:** 10.3390/polym17131837

**Published:** 2025-06-30

**Authors:** Liliane Nga, Benoit Ndiwe, Achille Bernard Biwole, Jean Jalin Eyinga Biwole, Mewoli Armel, Joseph Zobo Mfomo, Anélie Petrissans, Antonio Pizzi, Antonios N. Papadopoulos

**Affiliations:** 1 Laboratory of Forest Resources and Wood Valorization, Training Unit in Engineering Sciences, Post Graduate School of Fundamental and Applied Sciences, University of Douala, Douala P.O. Box 1872, Cameroon; lilianenga34@gmail.com (L.N.); achille.biwole@gmail.com (A.B.B.); eyinga.jj@gmail.com (J.J.E.B.); zobo_mfomo@yahoo.fr (J.Z.M.); 2 Laboratory of Mechanics, Training Unit in Engineering Sciences, Post Graduate School of Fundamental and Applied Sciences, University of Douala, Douala P.O. Box 2701, Cameroon; 3 Department of Industrial and Mechanical Engineering, National Advanced School of Engineering, University of Yaoundé 1, Yaoundé P.O. Box. 8390, Cameroon; mewoliarmel@yahoo.fr; 4 Laboratory of Studies and Research on Wood Material (LERMAB), University of Lorraine, 54000 Nancy, France; anelie.petrissans@univ-lorraine.fr (A.P.); antonio.pizzi@univ-lorraine.fr (A.P.); 5 Laboratory of Wood Science—Chemistry & Technology, Department of Natural Environment & Climate Resilience, Democritus University of Thrace, 1 km Drama-Mikrochoriou, 66100 Drama, Greece

**Keywords:** tannins, tropical wood barks, bio-based resins, characteristics, applications

## Abstract

The use of renewable plant resources for the formulation of adhesives is increasingly promising, thanks to their availability at an affordable price and their high content of biomolecules such as polyphenols. The study of tannins therefore remains an active and ongoing area of research. This article presents a recent characterization of tannins extracted from the barks of four types of tropical trees (*Entandophragma candolei*, *Entandophragma cylindricum*, *Afzelia africana* and *Dacryodes klaineana*) and their application in the development of bioresins. Tannin extraction with hot water yielded between 25% and 40%. Tannin from *Entandophragma candolei* produced the highest yield. Chemical analysis confirmed the high presence of condensed tannins, with the identification of several new monomers in each tannin type, underlining their uniqueness. The most chemically stable tannins, *Dacryodes klaineana* and *Afzelia africana*, demonstrated their ability to withstand temperatures of 525 °C and 375 °C, respectively, with carbon residues of 45.05% and 43.18%. As for the resins, *Entandophragma candolei* tannin resin stood out for its thermal properties, notably a degradation temperature of 500 °C and a carbon residue rate of 36.72%. As for *E. cylindricum* resin, it boasted the highest modulus of elasticity (5268 MPa). Characterized tannins can be exploited in the technological sector.

## 1. Introduction

Faced with the environmental challenges posed by the intensive use of fossil fuels, many industrial sectors are making the search for materials of natural origin their priority [1]. Biopolymers, in particular, represent a promising alternative to petroleum-based polymers. According to the “Global Bioplastics and Biopolymers Market Report 2023”, the value of the international bioplastics and biopolymers market is expected to reach USD 27.3 billion by 2027 [2], reflecting strong interest in renewable materials [3,4]. Tannins, due to their availability, low ecological impact and attractive physicochemical properties, are gaining in popularity among exploitable natural resources [4,5]. Currently, these plant-derived polyphenols, present in various plant tissues such as bark, leaves, wood and fruit, are being put to good use in the design of bioresins used mainly in the production of particleboard and composite materials [6,7].

There are four main types of tannins: condensed tannins (CT), hydrolyzable tannins (HT), phlorotannins (PhT) and complex tannins [8]. Condensed tannins account for almost 90% of commercial production worldwide. These include flavan-3-ol oligomers such as catechin, epicatechin and gallocatechin. Thanks to their reactive structure, they offer adhesion performance comparable to that of traditional phenolic resins, while also boasting antioxidant, antifungal, antimicrobial and anticorrosion properties [7,8]. However, most current research is based on the use of purified commercial monomers, such as gallic acid, catechin or resorcinol, derived from controlled extraction processes. Although this method is effective in the laboratory, it remains costly and difficult to implement on an industrial scale [7,9,10].

Similarly, the potential of tropical plants, particularly in Central Africa, remains underutilized. With a forest area of 22,000 hectares representing 42% of its territory, Cameroon produces more than a million tonnes of lignocellulosic residues every year, mainly from the exploitation of its forests [7,8]. These residues, particularly bark, represent a significant potential source of tannins. Numerous studies have identified tannin-rich Cameroonian species such as *Aningeria superba*, *Ficus sycomorus*, *Vitellaria paradoxa* and *Paraberlinia bifoliolata* [11,12,13,14,15], but these studies have not yet led to any tangible applications in the development of bio-based resins.

Consequently, this research project aimed to identify new sources of tropical tannins from Cameroonian tree bark. Specific objectives included, on the one hand, identifying the chemical and thermal properties of these extracts and, on the other, formulating bioresins and assessing their mechanical and thermomechanical performance. This research differs from previous work in that it seeks to valorize existing and underexploited local biomass while suggesting an economically viable method for the manufacture of bio-based materials for the particleboard industry and ecological inhibitors.

## 2. Materials and Methods

### 2.1. Raw Materials

#### 2.1.1. Chemical Materials

NaOH, boiling point 120.0 °C, melting point 8.0 °C, density 1.3600 g/mL, purity 33%; NaHSO_3_ density 1.33, purity 42%, melting point 150 °C (Lerochem); NaHCO_3_ purity 99%, melting point 50 °C, boiling point 851 °C; distilled water.

#### 2.1.2. Ecological Hardening Agents

The *Acacia nilotica* hardener was used in the resin formulation. Initially, *Acacia nilotica* exudates were collected from the *Dacheka* forest, located in the far north region of Cameroon. After being dried at room temperature for two weeks, they became soluble powder through grinding and are termed hardeners. This process facilitates their packaging and incorporation into the formulation of various resins [16].

#### 2.1.3. Tannin Extraction

During November, bark samples were collected from four wood species (*Entandophragma cylindricum, Entandophragma candolei, Afzelia africana and Dacryodes klaineana*) at the logging and wood processing company TRANSBOIS, located in the industrial zone of Douala, in the Littoral Region of Cameroon. Bark collection was carried out selectively, with each tree species being packaged in 50 kg bags. Once collected, a drying process was implemented. The barks were individually air-dried at room temperature (28 °C) for a period of 7 days. Subsequently, they were reduced to very small particles. For the experiment, fine bark particles of each species, extracted from the anhydrous mass, were immersed in an aqueous solution. This solution contained 2% sodium bisulfite and 0.5% sodium bicarbonate. The solid liquid ratio was 1:6 (*v*/*v*). The solution was heated to a temperature of 60 °C and then constantly stirred for 4 h. For the study, the solution was filtered through a cotton cloth to obtain a filtered liquid whose color varied depending on the species. The recovered liquid fraction underwent an evaporation process at a temperature of 60 °C, leading to the formation of tannin crystals. For analysis and preservation, it is necessary to have an easy-to-use tannin powder. To achieve this, the crystals were ground into powder using a ceramic mortar [17]. The extraction yield was then calculated using Equation (1):

(1) $$percentage=\frac{m_{\tan nin}}{m_{barks}}\times 100$$

where $m_{\tan nin}$ is the mass of the extracted tannin powder and $m_{barks}$ is the mass of the bark powder taken for extraction.

### 2.2. Tannin Characterization

#### 2.2.1. ^13^C NMR Analysis

The concentrated tannin extracts (aqueous solution 47%) were analyzed using ^13^C NMR according to a previously reported method [18]. The liquid ^13^C NMR spectrum of the tannin extracts was obtained on a Bruker MSL 300 FT-NMR spectrometer (Bruker, Wissembourg, France). The chemical shifts were calculated with respect to (CH_3_)_3_Si(CH_2_)_3_SO_3_Na dissolved in D2O for NMR shift control. The spectra were taken at 62.90 MHz. The spectra (10,000 transients) were attained on a Bruker MSL300 FT-NMR spectrometer, at a frequency of 62.9 MHz. Chemical shifts were calculated relative to (CH_3_)_3_Si(CH_3_)_3_SO_3_Na in D2O, with a precision of 1 ppm. The relaxation delay was 5 s [19].

#### 2.2.2. MALDI-TOF Analysis

The samples were treated with a NaCl solution (1.5 µL of 0.1 M) in a methanol/water mixture (1:1) to increase ion formation and a drop was placed on the MALDI target (3 mm diameter) steel plate and dried. The samples and the matrix were then mixed in equal amounts and 1.5 µL of the resulting slurry was placed on the MALDI target and dried at 40 °C for 2 h before being analyzed. A matrix of 2,5-dihydroxy benzoic acid was used. Red phosphorous (500–3000 Da) was used as a reference for spectrum calibration. Finally, after evaporation of the solvent, the MALDI target was introduced into the spectrometer. The spectra were recorded on a KRATOS AXIMA Performance mass spectrometer from Shimadzu Biotech (Kratos Analytical Shimadzu Europe Ltd., Manchester, UK). The irradiation source was a pulsed nitrogen laser with a wavelength of 337 nm. The length of one laser pulse was 3 ns. Measurements were carried out using the following conditions: positive polarity, a linear flight path, 20 kV acceleration voltages and 100–150 pulses per spectrum. The delayed extraction technique was used applying delay times of 200–800 ns. The software MALDI-MS (Kratos Analytical Shimadzu Europe Ltd., Manchester, UK) was used for the data treatment. The oligomers appeared in the spectra either corresponding to their molecular weight or their molecular weight +23 Da of the Na^+^ ion derived from NaCl, used as an enhancer. The spectra precision was ±1 Da [20].

#### 2.2.3. Thermogravimetric Analysis of Tannin

In this study, the NETZSCH STA 449 F3 Jupiter analyzer was used to evaluate the thermal stability and decomposition of tannins. In this experimental protocol, a precise quantity of sample powder, with an exact mass of 20 ± 1 mg, was deposited on a platinum platform. Then, the sample was heated from 25 °C to 600 °C, at a heating rate of 20 K·min^−1^, under a nitrogen atmosphere, thus allowing optimal control of the thermodynamic conditions of the experiment [21].

### 2.3. Resin Characterization

#### 2.3.1. Resin Formulation

The resin was formulated according to the protocol described in [22], which stipulates that an aqueous solution was prepared by mixing 40% tannin powder extracted from each previously mentioned wood species with 40% water and 10% natural exudates of *Acacia nilotica*. The potential of hydrogen (pH) of the mixture was adjusted to 8 by introducing a 33% sodium hydroxide solution [23].

#### 2.3.2. Physical Characterization of Resins: Gel Time

Approximately 10 g of the formulated resin without the addition of a sodium hydroxide (NaOH) solution (33% NaOH) was introduced into a test tube and placed in a water bath, maintained at boiling temperature (100 °C) at normal atmospheric pressure. A metal spring was inserted into the test tube and moved rapidly up and down. The gel time was measured using a stopwatch. The test was performed three times and the average value is reported as in [24]. Then, the resin was formulated by adding a sodium hydroxide solution (33% NaOH) to adjust the different pH levels of the mixture [25].

#### 2.3.3. Thermogravimetric Analysis of Resins

For this study, the NETZSCH STA 449 F3 Jupiter analyzer was used to evaluate the thermal stability and decomposition of the formulated resins. In this experimental protocol, a precise quantity of sample powder, with an exact mass o 20 ± 1 mg, was deposited on a platinum platform. Then, the sample was heated from 25 °C to 600 °C, at a heating rate of 20 K·min^−1^, under a nitrogen atmosphere, thus allowing optimal control of the thermodynamic conditions of the experiment [26].

#### 2.3.4. Thermomechanical Analysis of the Resins

This method, aimed at characterizing resins, allows for the analysis of interactions between the polymer and various molecules. It also provides data regarding the rigidity of the resin as a function of temperature [10]. Sample preparation involves placing 25 milligrams of specifically prepared resin between two smooth plates measuring 21 millimeters in length, 6 millimeters in width and 1.1 millimeters in thickness. They are then fixed and inserted into a Mettler Toledo 40 Thermomechanical Analyzer (TMA) (MettlerToledo, Zurich, Switzerland). The beech–resin–beech sandwiches were subjected to non-isothermal tests, with a temperature range from 25 °C to 250 °C, at a heating rate of 10 °C/minute. The area covered by the adhesive on the panels was 200 g/m^2^. These mentioned plates were decorative beech wood veneers of medium density (0.750 g/cm^3^), with a thickness of 0.5 mm and a moisture content of 11% [27,28]. The samples underwent three-point bending tests over a span of 18 mm, with the application of a force cycle of 0.1/0.5 N and a force cycle of 12 s (6 s/6 s). The mechanical relationship between force and deformation was determined using the following equation,

$$E=\frac{L^{3}}{4bh^{3}}\times \frac{F}{\Delta_{fbois}-\Delta_{fadhesif}}$$

where *E* is Young’s modulus, *L* is the span length, *b* and *h* are the width and thickness of the specimen, respectively, *F* is the force exerted on the assembly and Δ*_f wood_* and Δ*_f adhesive_* are the deflections which have been proven constant and reproducible [5]. The tested formulations were those prepared with the different extracted tannins.

## 3. Results and Discussion

### 3.1. Tannin Extraction Yield from Different Woods

Table 1 shows the yield of tannin extracts obtained. The study revealed that the yield of tannin extract obtained by the hot water maceration method varied between 25% and 40%. Indeed, SPL (35%) and KSP (40%) barks, belonging to the *Meliaceae* family, stood out for their remarkably high extraction yields. Next came DSS bark (33%), from the *Fabaceae* family and ADB (25%), a species belonging to the *Burseraceae* family. The variations observed can be attributed to the difference in value linked to the water content of the bark powder used for each type of wood. Water-rich materials represent a specific challenge during extraction, as they require a greater quantity of solvent than water-poor materials. In addition, the result can be influenced by the density of the wood and the thickness of the bark. DSS is characterized by its high density (0.90 to 1.15 g/cm^3^), but its bark is thinner than that of *Enthagrophragma* species, whose density varies between 0.69 g/cm^3^ (SPL) and 0.75 g/cm^3^ (KSP), with a bark thickness ranging from 4 to 8 mm, while ADB has the lowest density and a thin bark [29]. Research by Kouam et al. in 2012 demonstrated that the Entagrophragma genus contains flavonoids such as catechin and epicatechin, which are condensed tannins [30]. According to Konai in 2021, the extraction percentage of tannins from tropical species varies between 29% and 46% [23].

### 3.2. Chemical Structures of Molecules Identified in the Samples

**Figure 1:**
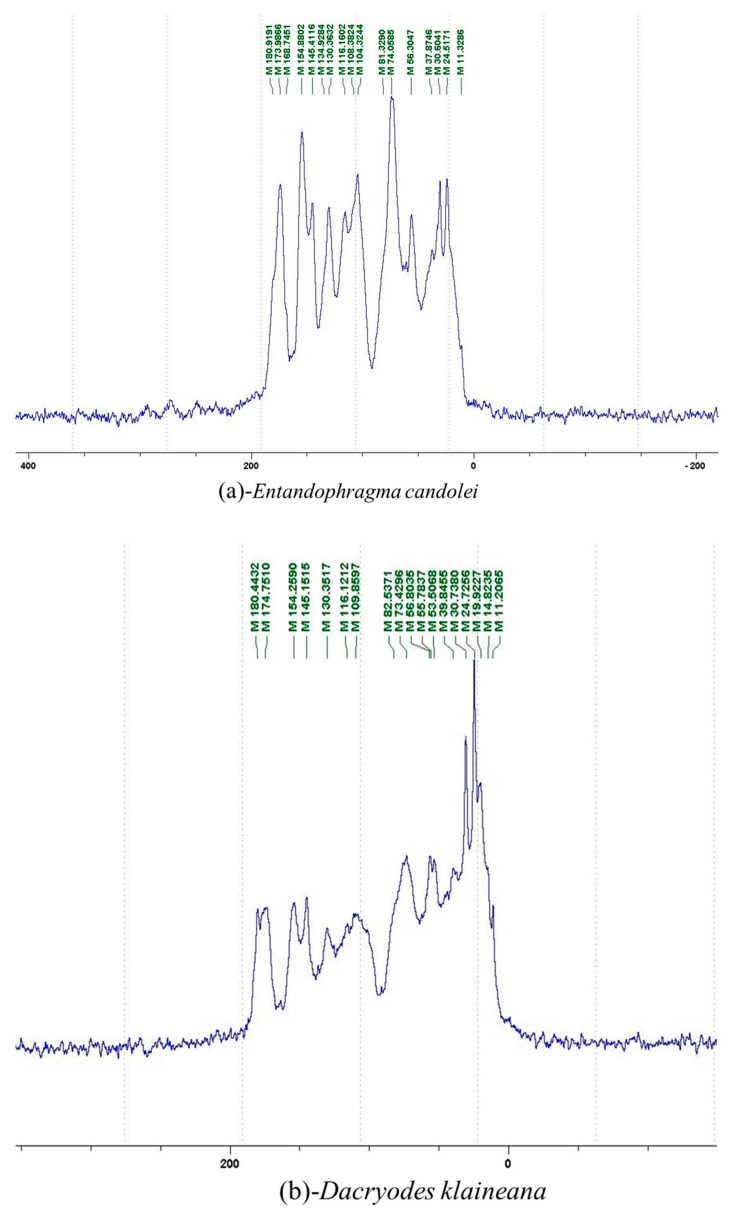

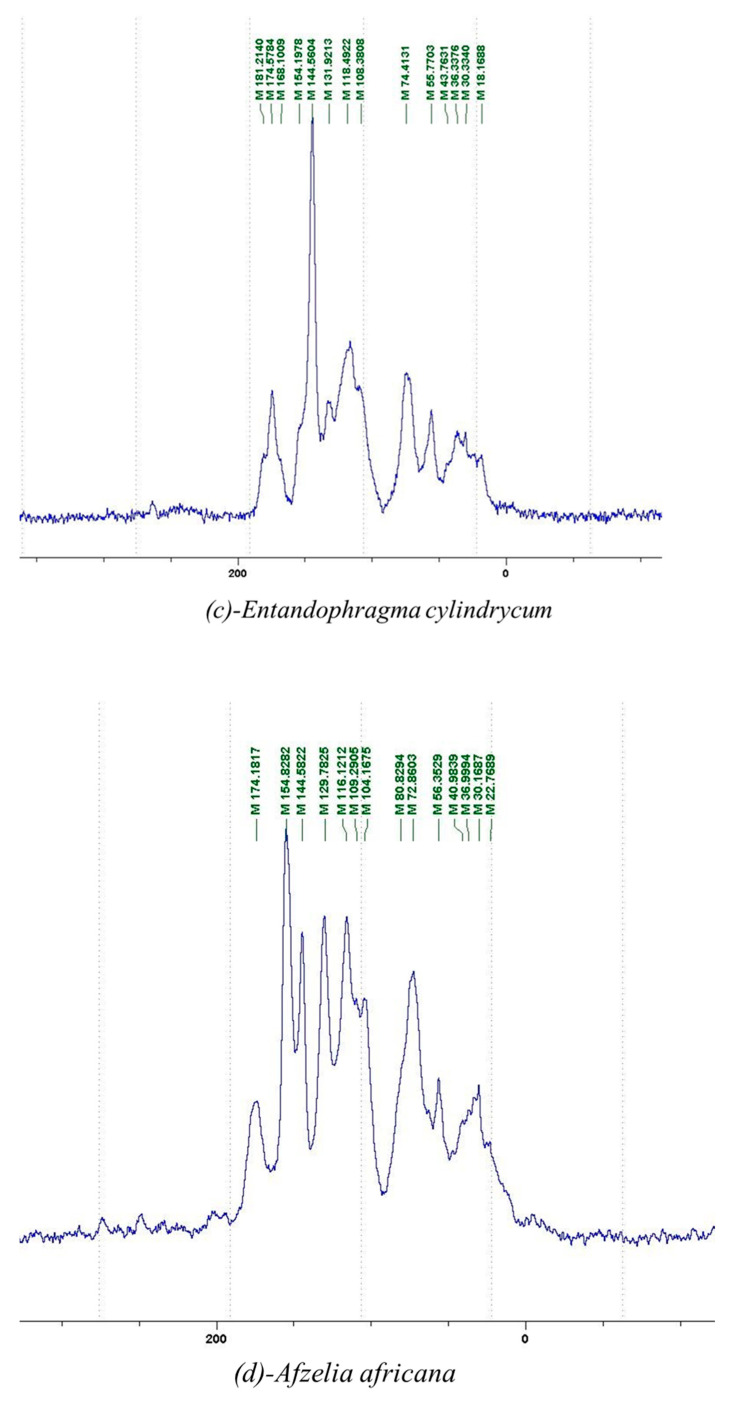
^13^C NMR spectra of the samples of the four analyzed tannins.

The ^13^C NMR spectra of tannin extracted from wood bark samples of *SPL*, *KSP*, *DSS* and *ADB* species were subjected to detailed analysis (Figure 1). This study highlighted the presence of various characteristic peaks identified in all examined tannins. The peak at 154 parts per million (ppm) is related to the vibrations of C9, C7, C5 of flavonoids, while the peaks at 144 ppm and 145 ppm represent the vibrations of the C3′ and C4′ rings in procyanidins [31,32]. The C1′ vibrations of flavonoids are found at peaks 129, 130 and 131 ppm at C4′ of prodelphinidins [14]. The 116 and 118 ppm peaks reflect the vibrations of the C5′ and C2′ bonds of procyanidins [14]. The C4–C8 interflavonoid linkage is identified between the 108 and 109 ppm peaks depending on the species. The 104 ppm peak observed in *KSP* and *DSS* is the signal related to the C6–C8 linkages of flavonoids [18]. The 80, 81 and 82 ppm peaks indicate the presence of C2 linkages of flavonoids in the *KSP, DSS* and *ADB* samples. The presence of the C-O-C function is revealed by peaks at 72 ppm in *DSS* and 74 ppm in *KSP*. The peaks at 24 and 11 ppm correspond respectively to free C4 and to the steric –CH2 and aliphatic –CH3 linkages of flavonoids [19,33].

The tannin spectrum of *KSP* (Figure 1a) showed peculiarities for certain peaks. The peak at 180 ppm is identified as corresponding to the –COOH function linked to a non-aromatic ring, while the peaks at 173 ppm and 168 ppm are respectively associated with the –C=O of an aliphatic and cyclic ester. The peak at 145 ppm is indicated by the C5 site of the furanic acid, prieuridine and azadiradrone rings. This observation is supported by the emergence of the peak at 134 ppm, which relates to the C2 vibration of these recognized compounds. The peak at 74 ppm concerns the C-O bond, linked to molecules such as aziridones and flavonoids. Furthermore, the existence of characteristic peaks of prieuridine is also observed. The peak at 37 ppm is linked to catechinic acid, while the peak at 30 ppm concerns the C6 bond of aziridone. Furthermore, tannin revealed new elements such as furanic acid, which belongs to the amino acid category, as well as prieuridine and aziridone, classified as alkaloids.

Furthermore, the analysis of the tannin spectrum of *SPL* (Figure 1c) allowed for the identification of specific peaks. In particular, the peak at 181 ppm is linked to the –COOH connection of strongly protonated acetic acid, derived from hemicellulose. Indeed, this band should appear at 178 ppm, but its identification at 181 ppm indicates degradation of hemicellulose during extraction. This variation may also be due to the presence of glucuronic or galacturonic acids, resulting from carbohydrate degradation, forming hemicellulose segments. Furthermore, the value of 147 ppm indicates the presence of –COOH, while 168 ppm is related to the –COOH of gallic acid. The peak at 74 ppm is characteristic of sugar oligomers, while the peak at 36 ppm corresponds to the carbon atom vibrations of Tau-cadinol, alpha-copaene and gamma-cadinene. The peak at 30 ppm is linked to the C4 of flavonoids and the CH2 groups of tau-cadinol, while the peak at 18 ppm represents the CH3 groups of tau-cadinol, alpha-copaene and gamma-cadinene.

Regarding the tannin profile of *DSS* (Figure 1d), the notable observed peaks are at 174 ppm; 56 ppm; 40 ppm; 36 ppm and 22 ppm. The peak at 174 ppm indicates the presence of substances such as eriodictyol and methoxyeriodiol, which are sterols. The peak at 56 ppm corresponds to the methyl group –OCH3 found in methoxyeriodictyol. The peak at 40.9 ppm is associated with the C3-H group of eriodictyol. The peaks at 36 ppm and 22.7 ppm indicate the presence of eriodictyol and methoxyeriodictyol, either alone or in combination with other flavonoids. Regarding the flavonoid tannin peaks, particularly those of C5, C7 and C9, which are generally located at 154 ppm, it was noted that the peak intensity is more pronounced. This is because it is an isolated peak for three carbon atoms, rather than the usual two peaks. The peak at 144 ppm indicates the highest concentration noted for C3′ and C4′ compounds. The C1 of flavonoids is associated with a value of 129.7 ppm. The peak at 116 ppm indicates the highest concentration of C2′ and C5′ for flavonoids such as catechin or fisetinidin (hemicellulose residues).

Furthermore, the 13C NMR spectrum analysis of tannin from *ADB* (Figure 1b) reveals the following distinct peaks: 180, 174, 73, 39, 27, 19 and 14 ppm. The peak at 180 ppm is associated with the –COOH group of protonated acid, originating from hemicellulose degradation. The peak at 174 ppm is linked to the C=O group of xanthones, while the peak at 73 ppm concerns carbohydrates, related to hemicellulose fragments. Finally, the peak at 55 ppm is associated with a –CH3 group linked to an O. The values of 39.8, 24.7, 19.9 and 14.8 ppm are associated with the compounds beta-amyrin, quercetin 3-O-alpha-L-rhamnoside, or the methyl ester of trihydroxy olean-12-en-28-oic acid. The identification of various signals related to phenolic hydroxyl groups confirms the condensed nature of each tannin examined [24].

### 3.3. Structural of Molecules from Different Tannin Samples

**Figure 2:**
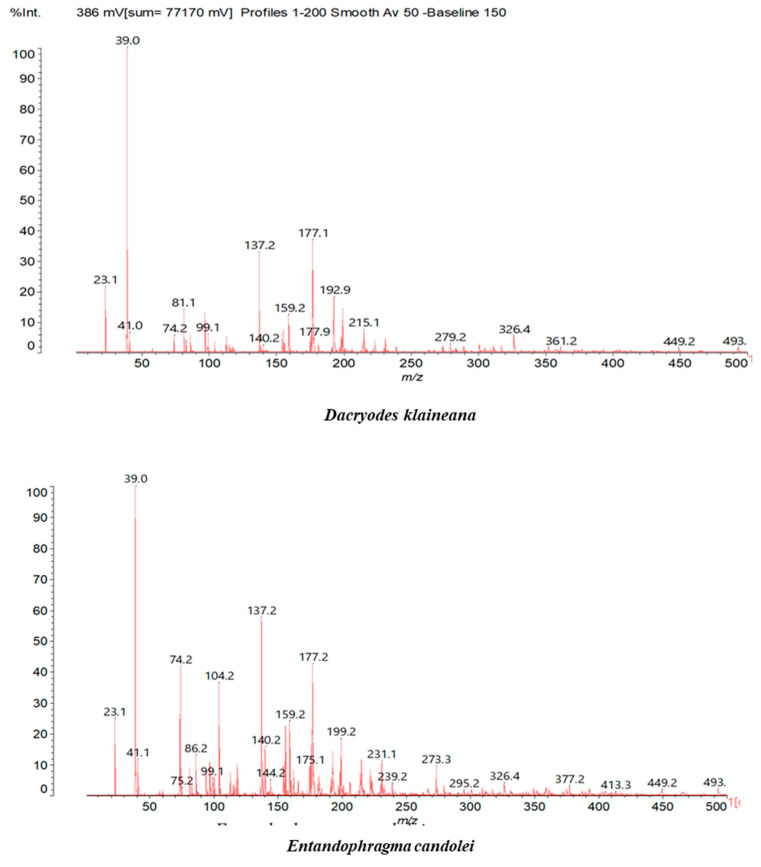

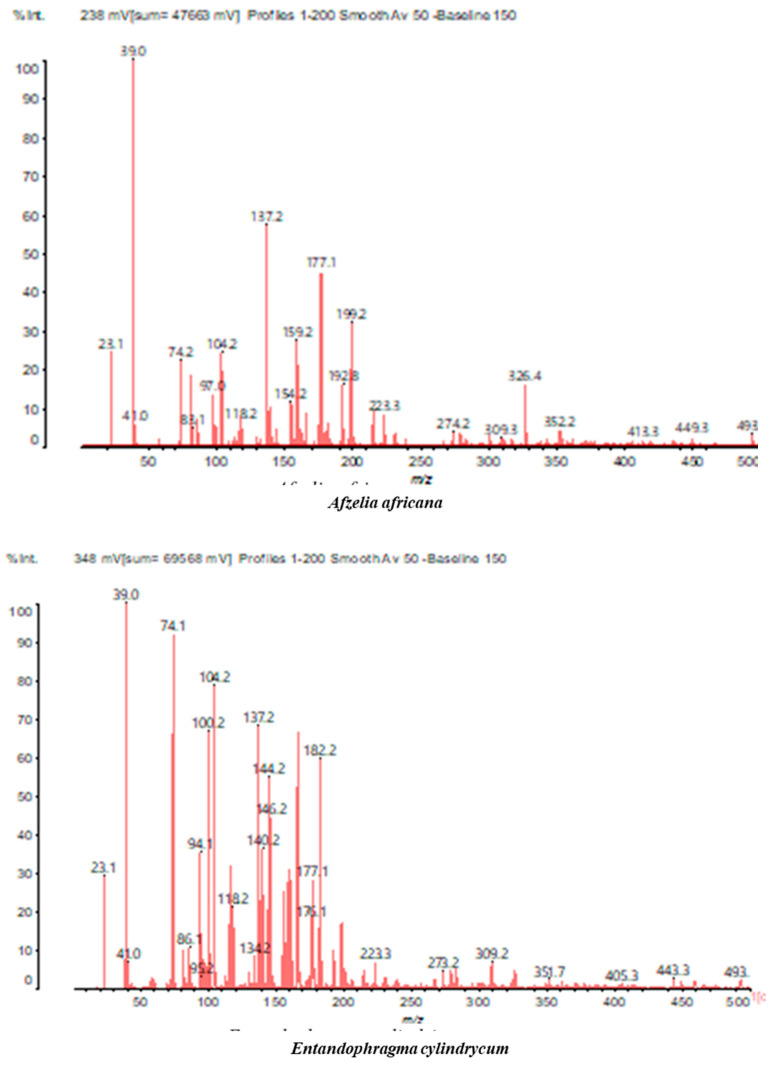
MALDI-TOF spectrum between 0 Da and 500 Da.

**Figure 3:**
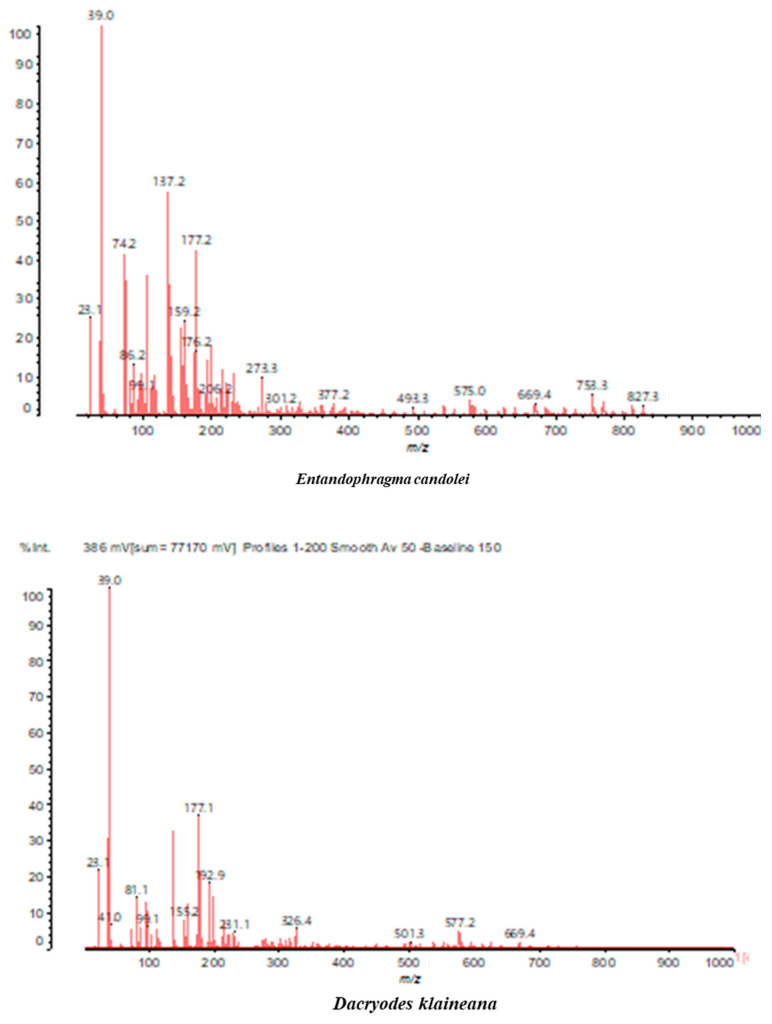

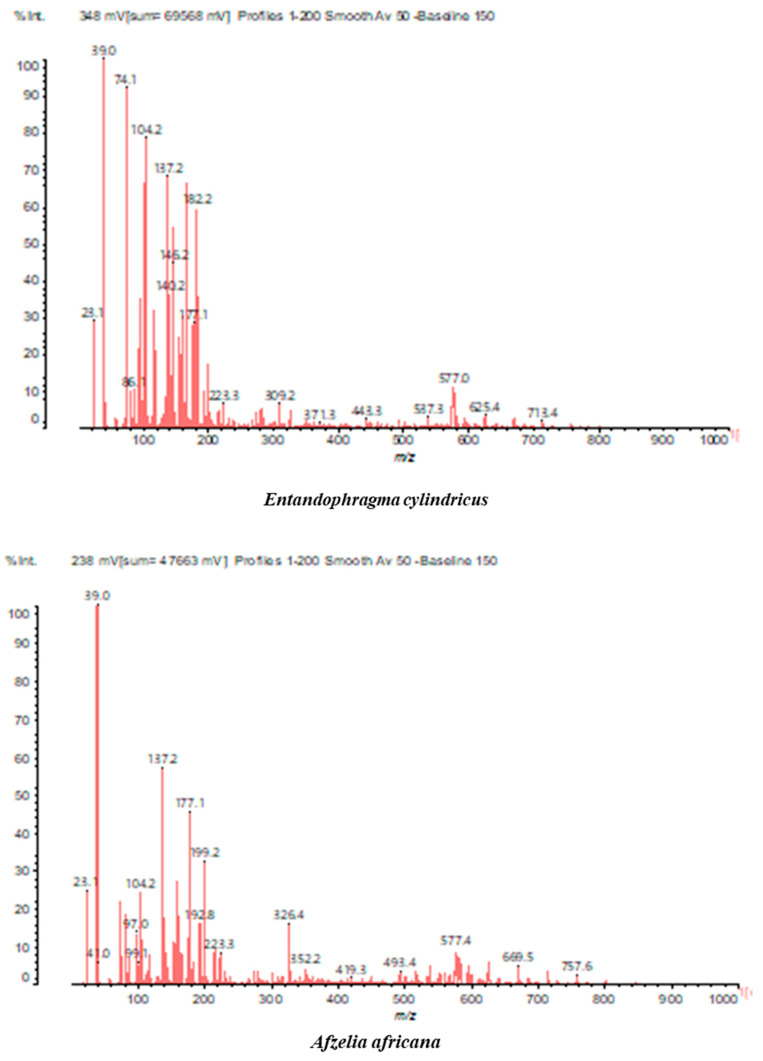

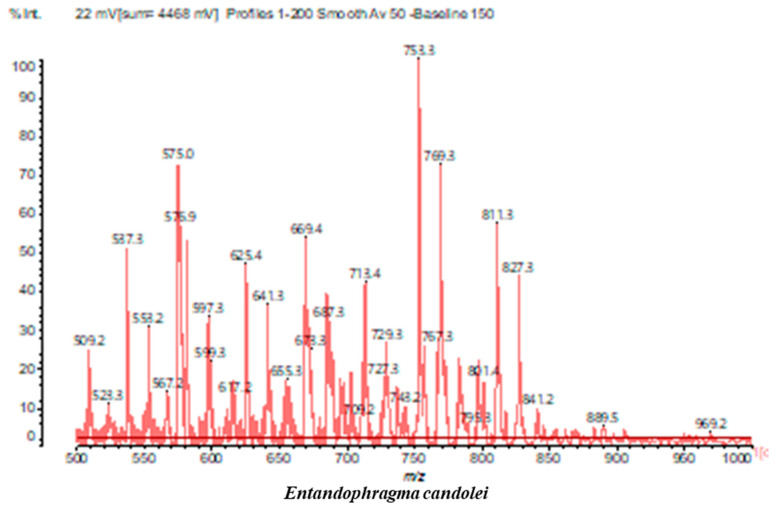
MALDI-TOF spectrum between 100 Da and 1000 Da.

**Figure 4:**
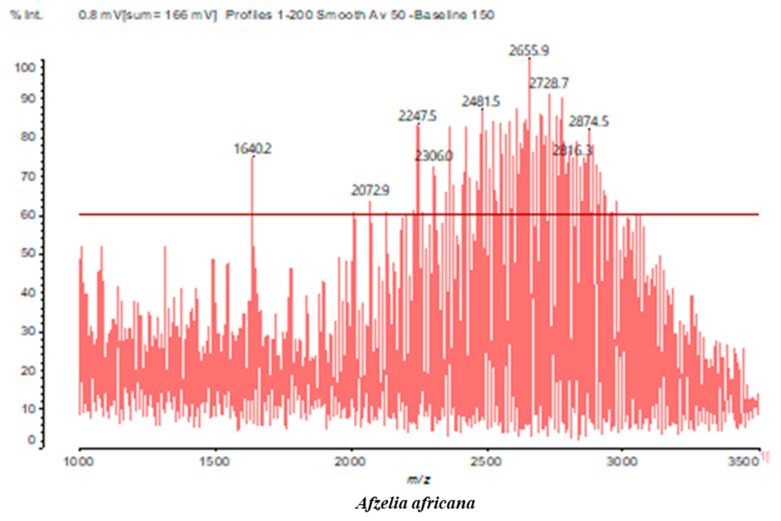

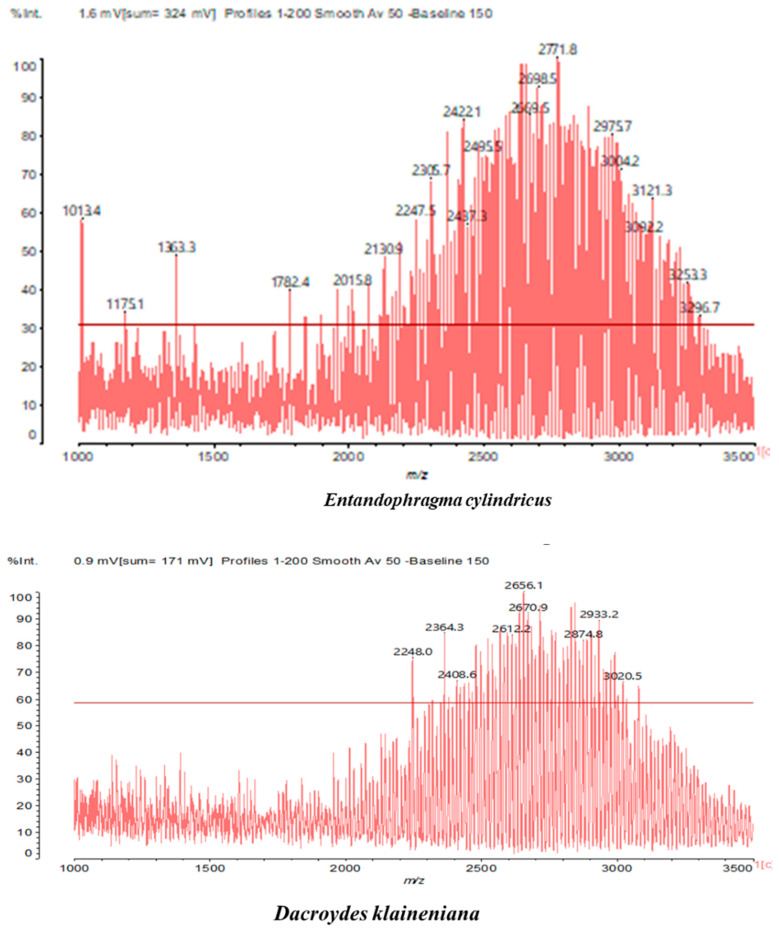

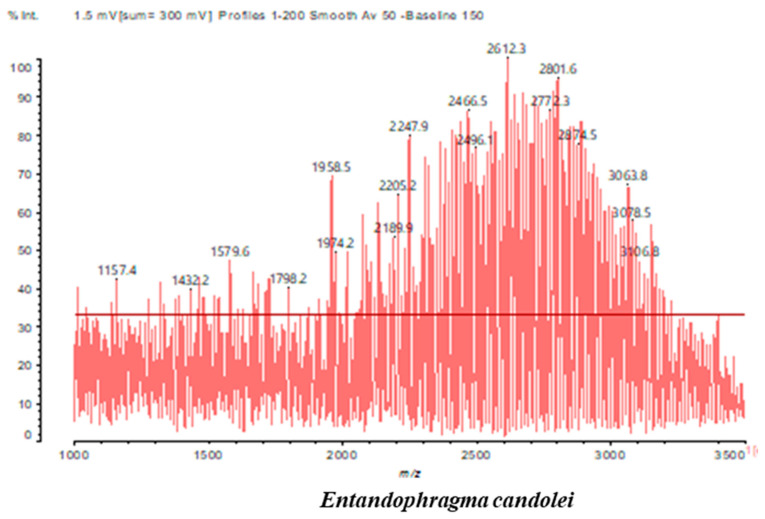
MALDI-TOF spectrum between 1000 Da and 3500 Da.

MALDI-TOF analyses of tannins extracted from SPL, KSP, DSS and ADB bark, as shown in Figure 2, Figure 3 and Figure 4, revealed the presence of oligomers already identified in other tannins. In addition, these analyses highlighted the presence of new condensed oligomers, as shown in Table 2.

In the *DSS* sample, the peak at 177 Da refers to a molecule Syringaldehyde fragment (**a**) with loss in 5xH found among the forest by-products in this sample (Bikoro Bi Athomo et al. 2020) [34]; 192 Da corresponds to protonated gallic acid (**b**) at 199 Da. 274 Da corresponds to the structure 14, 15-dehydrocrepenynic acid deprotonated [35]. The peak at 309 Da corresponds either to deprotonated eriodictyol bound to Na^+^, or to protonated anthraquinone bound to Na^+^ (the structures (**c**)), or 1-(2,5-dichlorophenyl)sulfonyl-4methylpiperazine [36]. 326 Da is protonated eriodictyol or a labile H of the –OH functional group that has been replaced by the –CH3 alkyl group (structure (**d**)). 352 Da = no Na^+^, deprotonated. This is a fragment of a flavonoid dimer like the structures at 193 Da above that were isolated in previous analyses (**e**). 449 Da corresponds to the catechin-3xH dimer linked to glucose [33]. The peaks 553, 575 and 577 Da correspond respectively to the robinetinidin-trihydroxyflavan +7H^+^ dimer and deprotonated gallocatechin (-H^+^ for 575 Da and -3H^+^ for 577 Da (**f**) [37]. The peaks at 581.5 and 584 Da correspond respectively to fisetinidin-eriodictyol and fisetinidin-catechin dimers, all bound to Na^+^. The 625 Da peak corresponds to the isoquercetin gallate +5H+ peak and 709 Da corresponds to the trimer of fisetinidin, gallocatechin and gallic acid [37]. 758 Da corresponds to the trimer of gallic acid, trihydroxyflavan and glucose hydrate. The peaks 772 Da protonated with Na^+^ and 801 Da without Na^+^ indicate the presence of a trihydroxyflavan and fisetinidin dimer (-8H^+^ for 772 Da) and (-2H^+^ for 801). 846 Da corresponds to a fisetinidin-catechin-eriodictyol trimer, deprotonated without Na^+^. 889 Da is a catechin trimer, bound to Na^+^.

For the *ADB* tannin, the following peaks were interpreted: 137 Da without Na^+^, 159 Da with Na^+^. These correspond to the following terpenes: (1) alpha-thujene; (2) alpha-pinene; (3) camphene; (4) sabinene; (5) beta-pinene; (6) alpha-phellandrene; (7) alpha-terpinene; (8) gamma-terpinene. 177 Da bound to Na^+^ corresponds to the following terpenic structures: (9) 1,8-cineole; (10) *cis*-sabinene hydrate; (11) terpinen-4-ol; (12) alpha-terpineol (**g**). The 192.8 Da peak is that of gallic acid bound to Na^+^. 196 Da corresponds to xanthone (**h**) in the absence of Na^+^. 326 Da = with Na^+^, either (**a**) deprotonated delphinidin, or (**b**) 9-(4-methoxyphenyl) xanthene (second most probable hypothesis) or both (**i**). 449 Da = with Na^+^, beta-amyrin; or without Na^+^ quercetin 3-O-alpha-L-rhamnoside (**j**). 509 Da = with Na^+^, deprotonated, methyl ester of 2,3,23-trihydroxyolean-12-en-28-oic acid new molecule (**k**). 575–577 Da = no Na^+^, sitosterol-3-O-beta-D-glucopyranoside sterol, (576 Da), protonated (577 Da) and deprotonated (575) and/or, or both catechin dimers, deprotonated (577) no Na^+^. 585 Da corresponds to a non-protonated catechin-fisetinidin dimer without Na^+^. The 595, 619 Da peaks protonated bound to Na^+^ correspond to the catechin-delphinidin dimer; 611 Da without Na^+^, protonated corresponds to the delphinidin-delphinidin dimer. 729Da corresponds to a fisetinidin dimer without Na^+^ bound to glucose molecules. 745 Da is a catechin-fisetinidin dimer bound to glucose molecules associated with Na^+^ and deprotonated. 760Da is a catechin dimer attached to glucose molecules. 921Da is a delphinidin-delphinidin-catechin trimer attached to Na^+^.

The following peaks allowed for the interpretation of the MALDI-TOF spectrum of *SPL*. The 192 Da peak corresponds to gallic acid bound to Na^+^ [7], 204 Da = no Na^+^, a small peak of alpha-copaene (1) or gamma-cadinene (2), or both mixed (**l**). 223 Da corresponds to tau-cadinol, protonated and bound to Na^+^ (**m**). The 273 Da peak is that of fisetinidin, without Na^+^. 309 Da without Na^+^, protonated delphinidin (307 Da), or with Na^+^, deprotonated eriodictyol (310) or both (**n**). 405 Da is the cadinol peak attached to glucose and Na^+^. The 443 Da peak is that of catechin gallate, without Na^+^. 493 Da corresponds to protonated delphinidin bound to glucose and Na^+^. 501 Da is the peak of fisetinidin esterified by tau cadinol without Na^+^, (**o**). 549 Da is a deprotonated fisetinidin-fisetinidin dimer without Na^+^. 577 Da is either a deprotonated delphinidin-fisetinidin dimer without Na^+^, or/and a catechin-catechin dimer. The 581, 595 Da peaks correspond respectively to the deprotonated catechin-fisetinidin dimer without Na^+^ and the delphinidin-catechin dimer without Na^+^ [7], protonated, or of fisetinidin-(glucose)_2_ without Na^+^. The 625–627 Da peak corresponds to delphinidin-(glucose)_2_ without Na^+^ [7]. 713 Da corresponds to the fisetinidin-fisetinidin-glucose structure, protonated without Na^+^ [7]. 757 Da corresponds to the catechin-delphinidin-glucose structure without Na^+^ [7]. 765 Da is delphinidin-fisetinidin-glucose, protonated bound to Na^+^. 889 Da corresponds to a catechin trimer, bound to Na^+^. 1175 Da is a glucose heptamer bound to Na^+^ or glucose-mannose, which is a hemicellulose fragment. 2015–2016 Da is a catechin heptamer, in the absence of Na^+^. 2306 Da is a catechin octamer, without Na^+^.

The following peaks caught our attention in the interpretation of the MALDI-TOF spectrum of *KSP*: the 199 Da peak is that of N-methyl 5-dihydroxypipecolic acid (**p**) [38]. 231 Da is attributed to deprotonated chalcone; 273 Da is fisetinidin, without Na^+^; 295 is attributed to the protonated catechin monomer. The 326 Da peak is that of protonated eriodictyol (**c**). 377 Da corresponds to dihydroxyflavan-3-(3,4-dihydroxy)-benzoate [30]. 413Da is attributed to the deprotonated chalcone dimer [7]; 449 Da corresponds to the dihydroxy pipecolic acid trimer [14]. (**q**). The peak at 493 corresponds to the quercetin-gallate monomer bound to Na^+^. 575 Da corresponds to the gallocatechin dimer –Na^+^, deprotonated. 669 Da is the pentamer of N-methyl 4-hydroxypipecolic (**r**) [39]. The peak has 493 Da = limonoid without Na^+^, deprotonated, calc. 493 Da (**s**). 509 Da = epoxyazadiradione (nimbinin) with Na^+^ (**t**), 523 Da = alpha-acetoxy-14beta,15beta epoxyazadiradirone, no Na^+^, deprotonated (**u**). 743 Da = no Na^+^; and 767 Da with Na^+^, may be a dimer between the limonoid azadirone and a gallocatechin assembled by a bond (**v**). 753 Da prieurianin (**w**). 801 Da = prieurianin acetate, with Na^+^ (**x**). 753 Da corresponds to catechin-delphinidin-glucose, without Na^+^, deprotonated [7]. The 827 Da peak is that of the deprotonated chalcone tetramer [7]. The peak at 1579 Da is that of fisetinidin/trihydroxyflavan, protonated [7].

### 3.4. Thermogravimetric Analysis of Tannins and Resins

**Figure 5:**
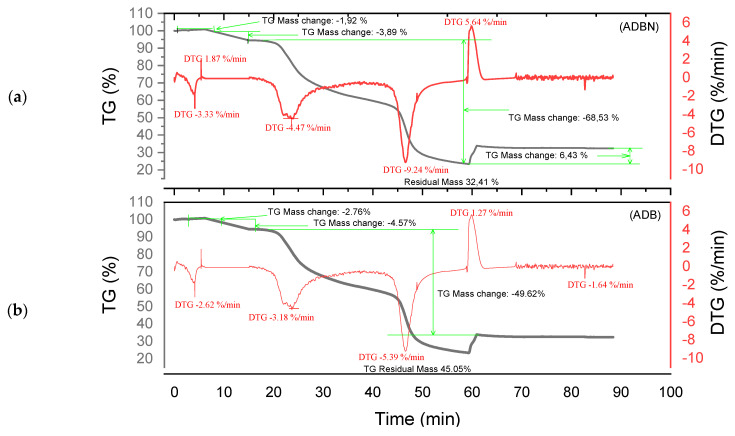
Curves TGA of ADBN and ADB (**a**,**b**).

**Figure 6:**
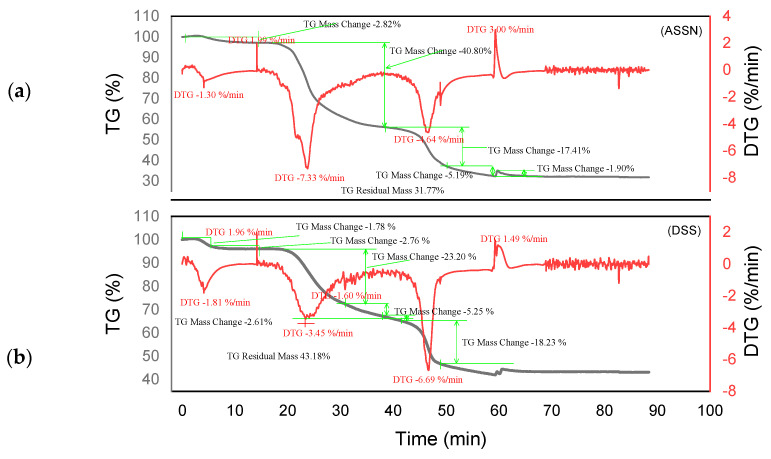
Curves TGA of DSSN and DSS (**a**,**b**).

**Figure 7:**
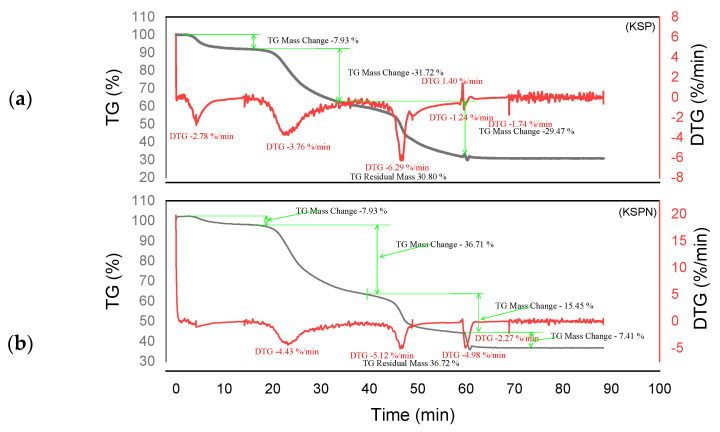
Curves TGA of KSP and KSPN (**a**,**b**).

**Figure 8:**
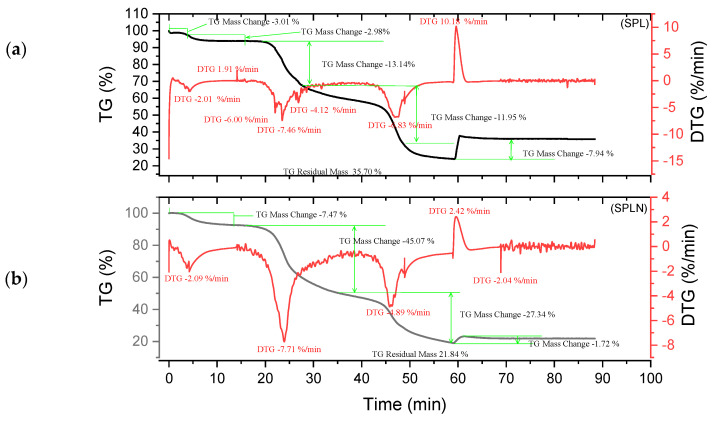
Curves TGA of SPL and SPLN (**a**,**b**).

Thermogram analysis was carried out on raw tannins ADB, DSS, KSP and SPL, as well as on tannin resins ADBN, DSSN, KSPN and SPLN under nitrogen atmosphere. Figure 5, Figure 6, Figure 7 and Figure 8 show several mass losses typical of thermal phenomena.

Different degradation patterns with numerous phases are revealed by the thermal study of ADB and DSS tannins (Figure 5a and Figure 6b) and their related resins (ADBN and DSSN) (Figure 5b and Figure 6a). At 95 °C, both ADB tannin and ADBN resin first lose water and volatiles [40,41]. At 275 °C, tiny molecules or heteropolysaccharides begin to degrade [42]. Due to its aromatic nature and mineral content, including silica [1], ADB tannin exhibits a third substantial degradation phase at 525 °C, which is linked to the breakdown of polyphenolic structures. At 600 °C, it yields a high char yield of 45.05% [43], whereas the ADBN resin degrades significantly at 600 °C, with a final residue of 32.41% at 625 °C, indicating a complex and thermally stable network of polymers. Similar to this, the DSSN resin has a final residue of 31.77% at 800 °C after initial moisture loss at 56.67 °C, volatilization of light compounds at 114.95 °C, main degradation at 241.63 °C and a second major breakdown at 371.19 °C, most likely involving aromatic structures [40,41]. With disintegration phases at 53.11 °C, 112.48 °C, 204.35 °C and 374.96 °C—the last of which shows the breakage of stable aromatic rings—DSS tannin exhibits a similar pattern. It concludes with a high residue of 43.18%, indicating that its dense aromatic component has given it significant thermal stability.

The tannin and resin samples reveal specific thermal behaviors with multi-phase degradation patterns when subjected to thermogravimetric analysis. At about 100 °C, all materials show early dehydration due to the removal of both bound and free water [44,45]. Similar decomposition profiles are shown by the tannin samples KSP and SPL. Early degradation starts at 200–280 °C, followed by main pyrolysis phases with primary DTG peaks at 320 °C, which involve the breakdown of polyphenolic structures and the cleavage of C–C/C–O bonds [46]. Decomposition is finished by 450–500 °C, with final residues of 30.80% (KSP) and 35.70% (SPL), indicating significant fixed carbon formation. Resin samples KSPN and SPLN, on the other hand, show more intricate degradation mechanisms with earlier onset temperatures (180–250 °C) and multiple distinct DTG peaks [43]. SPLN exhibits intense depolymerization at 290 °C, followed by secondary peaks at 380 °C and final decomposition extending to 480–500 °C [34]. KSPN shows primary degradation at 290 °C and secondary at 350 °C. The resin samples exhibit different residual behaviors. *SPL*N produces only 21.84% residue, showing more thorough combustion and less fixed carbon formation than tannin materials, whereas KSPN produces 36.72% residue, revealing its extensively cross-linked structure.

The thermal resistance of the materials under examination may vary depending on their maximum decomposition temperature and final residue. The most stable material is tannin ADB, which breaks down completely at 525 °C and leaves an important residue of 45.05%. The next most stable material is tannin DSS, which has a maximum breakdown temperature of 374.96 °C and produces a residue of 43.18%. This suggests a high presence of aromatic structures. Another characteristic that sets the KSPN resin apart is its considerable thermal stability (decomposition up to 500 °C, with a remainder of 36.72%). The resin ADBN shows a somewhat reduced residue (32.41%) despite a significant breakdown at 600 °C, indicating a polymer complex structure that is less rich in carbon. In contrast, compounds such as résine SPLN (residue 21.84%) or résine DSSN (residue 31.77%) exhibit more complete combustion or lower carbon densities, which makes them less heat-resistant than raw tannins. Therefore, it can be concluded that the tannins ADB and DSS are best suited to high-temperature applications, but SPLN has the worst thermal performance. Table 3 summarizes the performance of each of the materials studied in this research. These materials are more heat resistant than *Eucalyptus* and *Radiata pine* tannins, which start to break down at lower temperatures (165 °C and 150 °C, respectively) [1,17]. This is also the case with the hydrolyzable tannins that undergo Grenade referment breakdown at 149 °C and show the greatest degradation at 190 °C, leaving behind 36.4% residue [38]. While a percentage of residues can reach up to 55.29%, the tannins of *Eucalyptus Algerian* exhibit a peak of breakdown at 249.9 °C [47]. The thermal properties of the analyzed residues are superior to those developed with the tannin of *Alnus incana* and *Alnus glutinosa*, whose maximum degradation occurs at 132–133 °C, respectively [44].

### 3.5. Resin Gel Time

**Figure 9:**
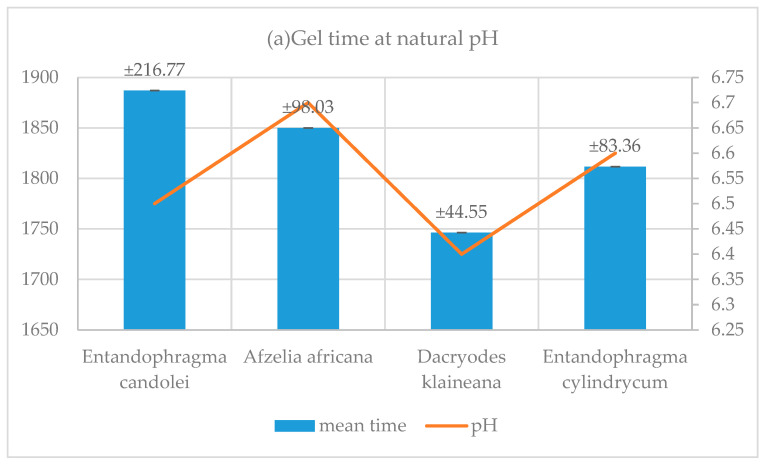

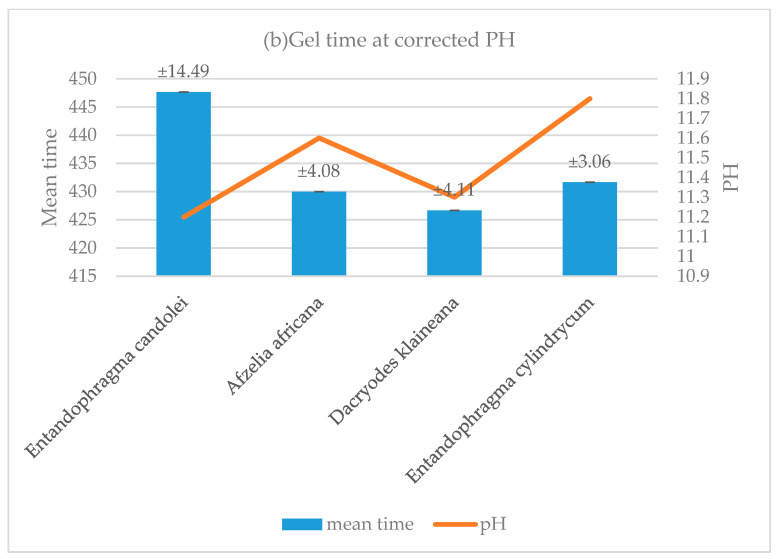
Influence of Ph on the curing time of resins.

The results in Table 4 and Table 5 and Figure 9 show that, for adhesive systems (KSPN), (DSSN), (ADBN) and (SPLN) at natural pH (6.4 and 6.7), gel time is significantly longer than when the pH is adjusted by adding NaOH (11.2–11.8). In the study carried out on resins and their use in the panel manufacturing process, it was observed that their shaping requires a pressing time of 600 s [45]. However, this time varies between 427 s and 448 s seconds when the pH is adjusted. This result is in line with the industrial panel manufacturing process. Two conclusions can be drawn from these results. Firstly, the adhesive systems (KSPN), (DSSN), (ADBN) and (SPLN) were shown to react and crosslink. Secondly, it has been established that these cross-links occur within a timeframe that meets industrial requirements. Although synthetic resins have a shorter curing time (urea-formaldehyde (UF) commercial 127 s) [42] than natural resins, the reactivity of the corrected resins formulated in this study remains interesting compared to that of others. This is the case for resins formulated with *Mimosa* tannin and hexamine (600 s) and *Mimosa* tannin and lignin in proportions of 90/10, 80/20 (623 s, 620 s) [46]. In addition, formaldehyde resins have been shown to have a very short gel time, of around 50 s at 100 °C for pure *Maritime pine* tannin cured with paraformaldehyde [48]. Compared with the longer gel time of the resin formulated in this study, we can therefore affirm that our resin does not contain a significant amount of aldehyde [19] and is therefore environmentally friendly.

### 3.6. Thermomechanical Analysis of the Resins

**Figure 10:**
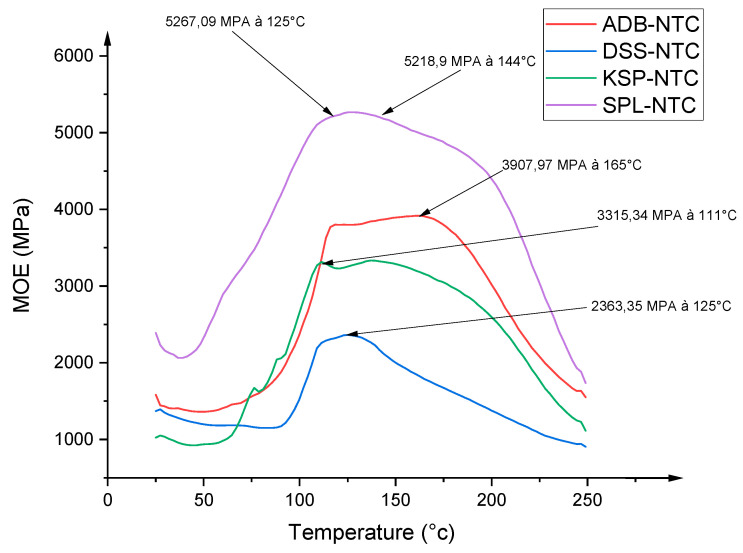
Superimposition of TMA spectra of the different tannins formulated with the *Acacia nilotica* hardener.

TMA results for the different resins formulated with each tannin and *Acacia nilotica* hardener are shown in Figure 10. Figure 10 shows four curves whose shape varies according to the reactivity of each tannin with the hardener. The resin formulated with SPLN tannin had the highest modulus of elasticity (5267 MPa) compared with the other formulations. This is followed by the ADBN tannin-based resin with a modulus of elasticity of (3907 MPa), then that formulated with KSPN tannin with a value of (3315 MPa) and finally that with DSSN tannin at (2363 MPa). This result can be justified by several factors, including the number of free reactive sites, the arrangement of the polymer chain, the degree of polymerization, the type of interflavanoid bonds, the natural variability of tannins and steric hindrance [17]. Covalent and ionic bonds can be formed between the various resin components. For these reasons, the MOE of the tannin adhesive may increase depending on the wood species. The presence of aromatic -OH functions may also be at the origin of this result. The presence of free hydroxyl groups makes tannin easily accessible and feasible for chemical modification [14,15]. Recent studies have shown that resins based on tannin as the main component and furfuryl alcohol are less effective. This is because furfuryl tannin resins interact with tannin, rather than being involved in self-condensation reactions. Three reaction mechanisms are possible: serial condensation, hydroxyl group interaction and furan ring rearrangement. Rearrangements lead to a cross-linking reaction [16,17]. Since the hardener used is furan-based, crosslinking is more effective with SPL tannin than with other tannins. However, the resins formulated and examined offer new prospects for the formulation of formaldehyde-free wood-based adhesives. The conclusions drawn concur with those of Osman, who characterized the tannin of *Acacia nilotica* spp. *nilotica* (*Ann*). His results show that the maximum Young’s modulus values are between 3300 and 3600 MPa [36]. In addition, the results obtained from this analysis are better than those of resins formulated with hexamine and tannin from *Sumac* (2282 MPa), *Pomegranate* (2100 MPa), *Mimosa* (2665 MPa), *Quebracho* (2464 MPa) and *Aleppo Pine* (2500 MPa) [19]. This highlights the high performance of the different resins formulated in this study.

### 3.7. Comparative Study of Characterized Tannins with Those from the Literature

Table 6 synthesizes the characteristics of tannins from tropical and commercial woods from recent studies, as well as those of the tannins that were the focus of this research. Tannins from tropical woods such as *Paraberlinia bifoliolata, Daniellia oliveri, Ficus sycomorus* or *Butyrospermum parkii* [6,29] are mainly condensed, with extraction rates ranging from 19% to 46%. Although some resins, such as *Butyrospermum parkii*, show remarkable mechanical strength (reaching up to 46,210 MPa) [6], information on their maximum decomposition temperature is frequently lacking, hampering the assessment of their thermal stability. In comparison, the tannins studied, ADB and DSS, show significant decomposition temperatures (525 °C and 375 °C, respectively) and significant residuals (45.05% and 43.18%), indicating a significant presence of aromatic structures and a remarkable potential for fixed carbon production. They are therefore considered more thermally stable than most tropical tannins.

Compared with commercial tannins such as *Pinus maritimus or Schinopsis balansae* [19,33], which have decomposition temperatures below 380 °C, these tannins stand out for their superior thermal stability. Furthermore, in comparison with recently analyzed tannins such as *Dacryodes klaineana* (525 °C) or *Entandophragma cylindricum* (280–310 °C), ADB tannin achieves comparable performance, while DSS stands out for its superior thermal and residual resistance. Thus, ADB and DSS tannins combine high thermal performance, structural stability and advanced application potential. These characteristics make them particularly suitable for demanding applications such as ecological resins, heat-resistant adhesives and corrosion inhibitors.

## 4. Conclusions

The objective of this research was to identify the characteristics of the tannins and resins obtained by macerating the barks of four tropical tree species, *Entandophragma candolei*, *Entandophragma cylindricum*, *Afzelia africana* and *Dacryodes klaineana*, in hot water. This study’s goal was to investigate the potential uses of these technologies in the engineering field. Consequently, we were able to identify the chemical and thermal properties of tannins while also figuring out the thermomechanical and physical properties of the resins made from each type of tannin. The RMN13C and MALDI-TOF investigations of the various tannins revealed their condensed nature as well as the identification of new oligomers. The most chemically stable tannins, ADB and DSS, were shown to withstand temperatures of 525 °C and 375 °C, respectively, with carbon residues of 45.05% and 43.18%. Regarding resins, the KSPN resin stands out due to its thermal characteristics, which include a degradation temperature of 500 °C and a carbon residue of 36.72%. By using NaOH to cool the gel, it is possible to achieve a pressure time below 600 s, which is in line with industrial panel pressuring procedures. The TMA analysis showed that the SPLN residue had the most significant MOE of 5267MPa. These characteristics make them particularly suitable for demanding applications such as ecological resins, heat-resistant adhesives and corrosion inhibitors. It is essential to experiment and optimize the formulations while switching up the hardeners in order to enhance and improve the results.

## Figures and Tables

**Table 1:** Yield of the tannin extract obtained.

Tannin Barks of	Yield (%)	References
SPL	35 ± 0.27	35 [12]
KSP	40 ± 2	35 [12]
DSS	33 ± 0.27	29 [12]
ADB	25 ± 1	40 [12]

**Table 2:** Different monomers identified in tannins extracted from tropical wood bark.

Species of Bark	New Compounds Identified by MALDI-TOF/MS	
***Afzelia africana (DSS)***	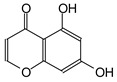 And 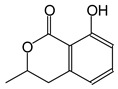	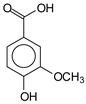
	(**a**)	(**b**)
	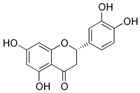 Or 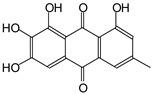	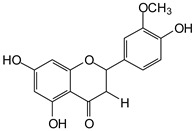
	(**c**)	(**d**)
	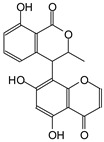	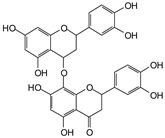
	(**e**)	(**f**)
***Dacryodes klaineana (ADB)***	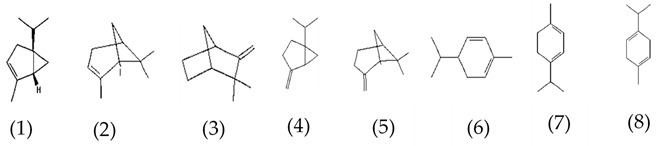	
	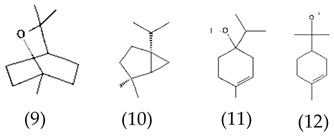	
	(**g**)	
	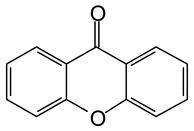	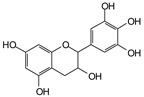 Or 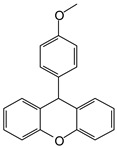
	(**h**)	(**i**)
	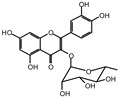 Or/And 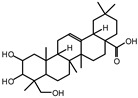	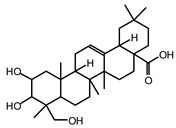
	(**j**)	(**k**)
***Entandophragma cylindricum (SPL)***	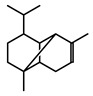 Or 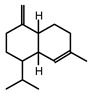	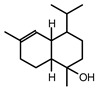
	1 2	
	(**l**)	(**m**)
	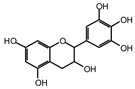 Or 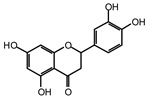	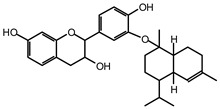
	(**n**)	(**o**)
***Entandophragma candolei (KSP)***	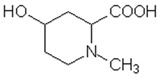	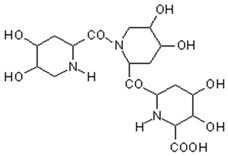
	(**p**)	(**q**)
	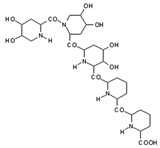 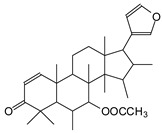	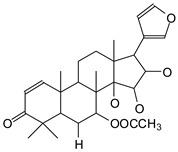
	(**r**) (**s**)	(**t**)
	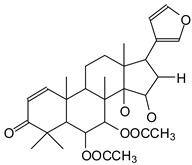	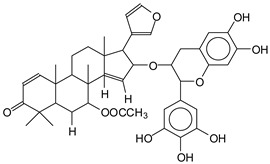
	(**u**)	(**v**)
	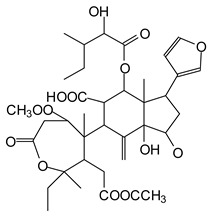	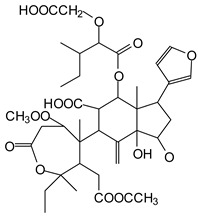
	(**w**)	(**x**)

**Table 3:** Thermal properties of the tannins and resins studied.

Material	Maximum Degradation Temperature (°C)	Final Residue (%)	Thermal Stability
***ADB***	**525**	**45.05**	Very stable
***DSS***	**374.96**	43.18	Very stable
***KSPN***	**500**	36.72	Stable
***ADB*** ***N***	**600**	32.41	Less stable
***DSS*** ***N***	**371.19**	31.77	Less stable
***KSP***	**500**	30.80	Unstable
***SPL***	**500**	30.80	Unstable
***SPL*** ***N***	**500**	**21.84**	Very unstable

**Table 4:** Measurement of resin gel time at natural pH.

Trials	1	2	3	Mean Time	Standard Deviation	pH
Species	Time in Second(s)					
*Entandophragma candolei*	1787	2188	1686	1887	± 216.77	6.5
*Afzelia africana*	1810	1985	1755	1850	± 98.03	6.7
*Dacryodes klaineana*	1692	1746	1802	1746.33	± 44.55	6.4
*Entandophragma cylindrycum*	1929	1756	1750	1811.67	± 83.36	6.6

**Table 5:** Measurement of resin gel time after addition of 33% concentrated sodium hydroxide.

Trials	1	2	3	Mean Time	Standard Deviation	pH
Wood Species	Time in Second (s)					
***Entandophragma candolei***	440	435	468	447.67	±14.49	11.2
***Afzelia africana***	430	425	435	430	±4.08	11.6
***Dacryodes klaineana***	432	426	422	426.67	±4.11	11.3
***Entandophragma cylindrycum***	429	436	430	431.67	±3.06	11.8

**Table 6:** Comparative study of characterized tannins with those from literature.

Species	Part of the Tree Used	Nature of Tannin	Extraction Yield (%)	Extraction Method	Maximum Temperature (Tmax, °C)	Thermomechanical Analysis of the Resin (MPa)	Gel Time	Applications	References
	**Tannins from Tropical Woods**								
***Paraberlinia bifoliolata***	Bark	Condensed	35%	Hot extraction in aqueous solution	-	4840	-	Adhesives for panels and corrosion inhibitors	[15]
***Aningre (Aningeria spp)***	Bark	Condensed	19%	Hot extraction in aqueous solution	-	1191	-	Resins for particleboard	[10]
***Piptadeniastrum africanum***	Bark	Condensed	-	Hot extraction in aqueous solution	-	3909	660 s	Adhesives for fiberboard	[7]
***Ficus sycomorus***	Barks	Condensed	46%	Hot extraction in aqueous solution	-	7050	600 s	Adhesives for fiberboard	[33]
***Butyrospermum parkii***	Barks	Condensed	40%	Hot extraction in aqueous solution	-	46,210	701 s	Adhesives for fiberboard	[33]
***Azadirachta indica***	Barks	Condensed	35%.	Hot extraction in aqueous solution	-	2650	762 s	Adhesives for fiberboard	[33]
***Ficus platyphylla***	Barks	Condensed	-	Hot extraction in aqueous solution		2091	-	Adhesives for particleboard	[12]
***Vitellaria paradoxa***	Barks	Condensed	-	Hot extraction in aqueous solution		1989	-	Adhesives for particleboard	[12]
***Cissus dinklagei***	Barks	Condensed		Hot extraction in aqueous zolution	300 °C	3825	-	Adhesives for particleboard	[14]
***Aningeria altissima***	Barks	Condensed	25.52%	Hot Extraction in Aqueous Solution	325 °C	5491.77	840–1201 s	Adhesives for Fiberboard	[13]
**Commercial tannins**									
***Pinus maritimus***	Bark	Polylavonoide tannin	-	Hot extraction in aqueous solution	-	2770305032503500	39–585 s	Adhesives for particleboard	[35,36]
***Schinopsis balancae***	Commercialized	Polylavonoide tannin		Industrial	-	-	238 s	Adhesives for particleboard	[24]
***Alnus incana***	Barks			Butanol–HCl method	132 °C	-		Adhesives for particleboard	[42]
***Alnus glutinosa***	Barks			Butanol–HCl method	133 °C	-		Adhesives for particleboard	[42]
**Studied tannins**									
***Entandophragma candolei***	Bark	Condensed	40%	Hot extraction in aqueous solution	500 °C	3315	448 s		
***Entandophragma cylindricum***	Bark	Condensed	35%	Hot extraction in aqueous solution	500 °C	5267	431 s		
***Afzelia africana***	Bark	Condensed	33%	Hot extraction in aqueous solution	374.96 °C	2363	430 s		
***Dacryodes klaineana***	Bark	Condensed	25%	Hot extraction in aqueous solution	525 °C	3907	427 s		

## Data Availability

The original contributions presented in this study are included in the article. Further inquiries can be directed to the corresponding authors.
